# Supplementary material for: Elucidating the Ability of CGRP to Modulate Microvascular Events in Mouse Skin
Source: Int J Mol Sci. 2022 Oct 13;23(20):12246. doi: 10.3390/ijms232012246 (PMC9602655; doi:10.3390/ijms232012246)
Supplement: Supplementary file 1 [file ijms-23-12246-s001.zip › IJMS Supplementary figures and legends.pdf]

Supplementary figure S1

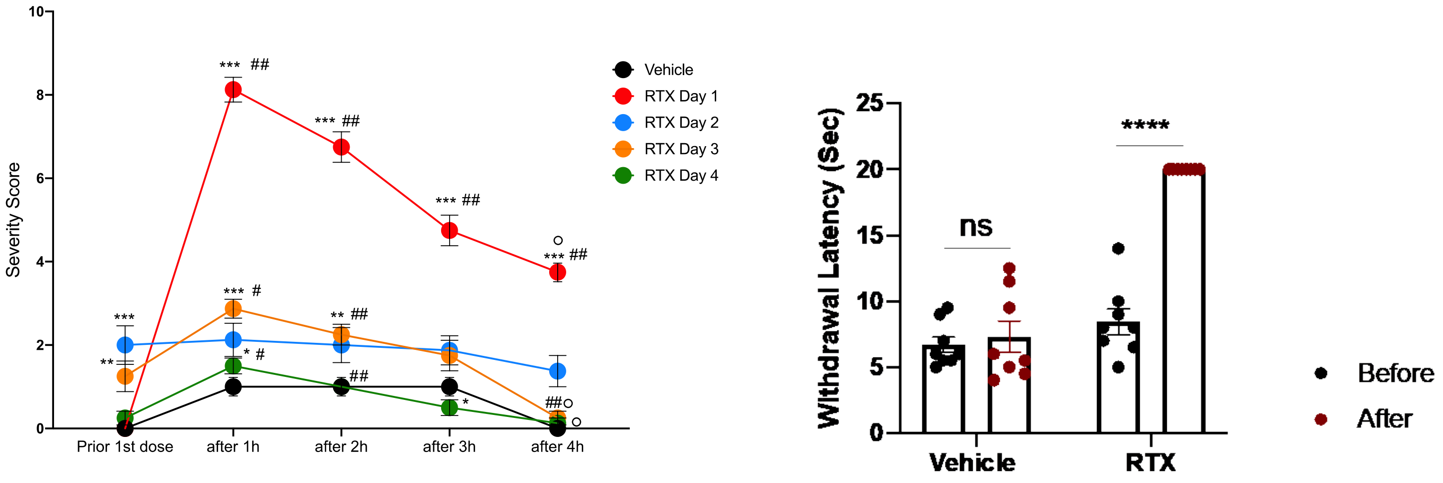

Supplementary Figure S1. The effect of the sensory nerve depleting agent RTX.
The figure shows (a) severity scoring by observation for 4 h after vehicle (10 % Tween-80, 10 % ethanol, 80 % saline) or RTX (0.3 mg/kg s.c.) on each of the 4 days. The parameters for the severity scoring include appearance, natural behaviour and provoked behaviour. All parameters were scored from zero to three with zero being a normal response and three being a severe response. Results are shown as mean ± SEM (n=8). Two-way ANOVA with Bonferroni's post hoc test. p*<0.05, p**<0.01 and p***<0.001 are compared to vehicle's respective timepoint. $p^{\#}<0.05$ and $p^{\#\#}<0.01$ are compared to respective baseline. o= p<0.0001 is compared to 'RTX after 1h' timepoint. (b) shows withdrawal latency (nociceptive behaviour) from a hot plate (55°C). A cut off time of 20 sec was used, for those that showed no response. Results are shown as mean ± SEM (n=8). Two-way ANOVA followed by Bonferroni's post hoc test. p****<0.0001 is compared to before RTX treatment.

Supplementary figure S2

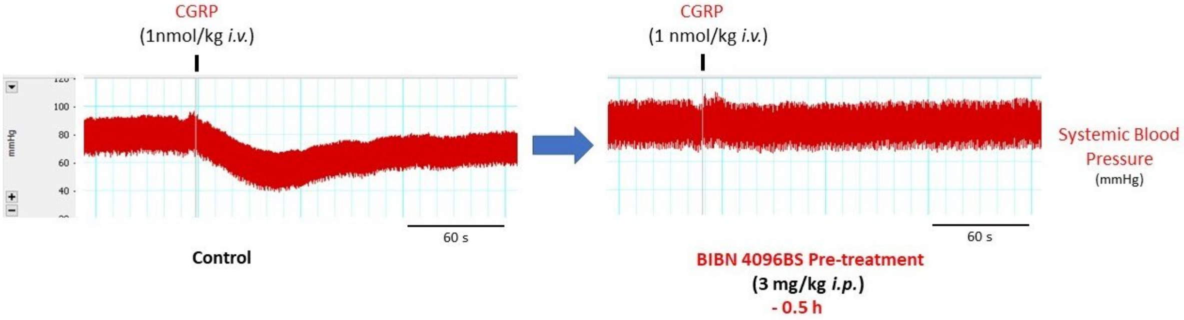

Supplementary Figure S2. BIBN4096 Blockade of CGRP hypotensive effect after carotid artery canulation.

(a) Blood pressure (mmHg) measured via carotid artery cannulation in a single anaesthetized mouse following CGRP (1 nmol/kg i.v.). (b) Systemic blood pressure (mmHg) measured in the carotid artery following CGRP 1 nmol/kg i.v.) in a mouse pre-treated with BIBN4096 (3 mg/kg i.p.) 30 min beforehand.

Supplementary figure S3

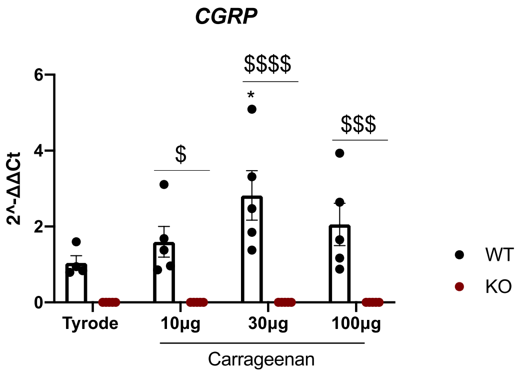

Supplementary Figure S3. CGRP mRNA expression (DRGs) after carrageenan treatment (i.d.) over 4 h (WT vs αCGRPKO mice).

CGRP gene expression in DRGs samples collected 4 h after Tyrode and Carrageenan (10µg, 30µg and 100µg) were injected i.d. in WT and αCGRPKO mouse dorsal skin. Results are shown as mean ± SEM (n= 4-5). Two-Way ANOVA followed by Bonferroni's post hoc test. p*<0.05 is compared to Tyrode. $p^{\$}<0.05$, $p^{\$\$\$}<0.001$ and $p^{\$\$\$\$}<0.0001$ are compared to WT Carrageenan.
